# Lambda Light Chain Cast Nephropathy Caused by Splenic Marginal Zone Lymphoma

**DOI:** 10.7759/cureus.75973

**Published:** 2024-12-18

**Authors:** Joyce H Gu, Nicholas Smothermon, Jusnimrat Kohli, Mark Samarneh

**Affiliations:** 1 Medicine, Lake Erie College of Osteopathic Medicine, Greensburg, USA; 2 Internal Medicine, St. John's Riverside Hospital, Yonkers, USA; 3 Internal Medicine/Nephrology, Riverside Health System, Yonkers, USA

**Keywords:** cast nephropathy, lambda light chain, light cast chain nephropathy, marginal zone lymphoma mzl, splenic marginal zone lymphoma

## Abstract

We report an interesting case of an elderly male patient with splenic marginal zone lymphoma with a monoclonal cluster of differentiation (CD)5+ B cells. The patient developed signs of renal injury and was evaluated via serum protein electrophoresis and immunofixation electrophoresis, which revealed monoclonal gammopathy with elevated free lambda light chain. However, a bone biopsy ruled out a diagnosis of multiple myeloma. Upon renal biopsy, the patient was diagnosed with lambda light chain cast nephropathy. However, the patient's condition further deteriorated, and the patient eventually expired. This case highlights light chain cast nephropathy as a rare yet serious complication of lymphomas and, in particular, splenic marginal zone lymphoma.

## Introduction

Immunoglobulins, also known as antibodies, are a critical component of our adaptive immune system that binds and neutralizes antigens. However, when immunoglobulins or their constituent components, such as light chains, are overproduced, for example, in the setting of hematologic cancers such as lymphomas, these molecules can deposit in various tissues and cause severe inflammation and injury. Because free light chains are predominantly filtered by the kidney, the kidneys are especially prone to injury in these conditions. In particular, light chain cast nephropathy refers to renal injury incited by the deposition of immunoglobulin light chain casts in renal tubules. These light chain casts interact with mesangial cells and cause renal inflammation and injury, eventually leading to acute kidney injury and acute renal failure [1]. Diagnosis is predominantly based on the identification of light chain deposition in kidney biopsy specimens via immunofluorescence imaging [1]. The prognosis for light chain cast nephropathy is poor, with 32% of patients expiring within 18 months, and there is currently no standard treatment for light chain cast nephropathy [1].

Light chain cast nephropathy is most often associated with plasma cell dyscrasias, and up to 50% of cases of light chain cast nephropathy are diagnosed as secondary to lymphoproliferative diseases [1]. In particular, light chain cast nephropathy is a defining feature of multiple myeloma (aka "myeloma kidney"). However, much more rarely, light chain cast nephropathy can also be associated with other B cell lymphomas [2], and there have been case reports that report light chain cast nephropathy associated with lymphomas [3] such as lymphoplasmacytic lymphoma [2,4-8], plasmablastic lymphoma [2,9], and diffuse large B cell lymphoma [10]. Here, we describe an unusual case of lambda light chain cast nephropathy caused by a splenic marginal zone lymphoma (MZL), which is rarely reported in the literature.

## Case presentation

The patient is a 73-year-old man who was admitted to the emergency department due to a one-month history of decreased appetite, weight loss of 18 pounds, fatigue, headaches, and a sore throat in June 2014. At admission, the patient is normotensive and normothermic, with normal pulse rate, respirations, and oxygen saturation. A CBC (Table 1) revealed elevated WBC count and mild anemia, raising suspicion of possible blood dyscrasia. A comprehensive metabolic panel (CMP) was notable for elevated blood urea nitrogen (BUN) at 24 mg/dL (7-18 mg/dL) and creatinine at 1.4 mg/dL (normal range 0.55-1.3 mg/dL).

**Table 1:** Complete blood count with differential, June 12, 2014. Hct: hematocrit; Hgb: hemoglobin; MCHC: mean corpuscular hemoglobin concentration; MCV: mean corpuscle volume; MPV: mean platelet volume; RBC: red blood cell count; RDW: red cell distribution width; WBC: white blood cell count

	Patient value	Normal range
WBC	15.6 K/mm^3^	4.0-10.0
RBC	4.86 M/mm^3^	4.00-5.60
Hgb	11.9 mg/dL	11.7-16.9
Hct	36.20%	35.4-49
MCV	74.5 fL	80-96
MCHC	32.8 g/dL	32.0-35.9
RDW	16.60%	11.9-15.9
Platelets	150 K/mm^3^	134-434
MPV	6.8 fL	7.5-11.1
Neutrophils	29%	42.8-82.8
Lymphocytes	55%	8-40
Monocytes	15%	3.8-10.2
Eosinophils	0%	0-4.5
Basophils	1%	0-2.0

**Figure 1:**
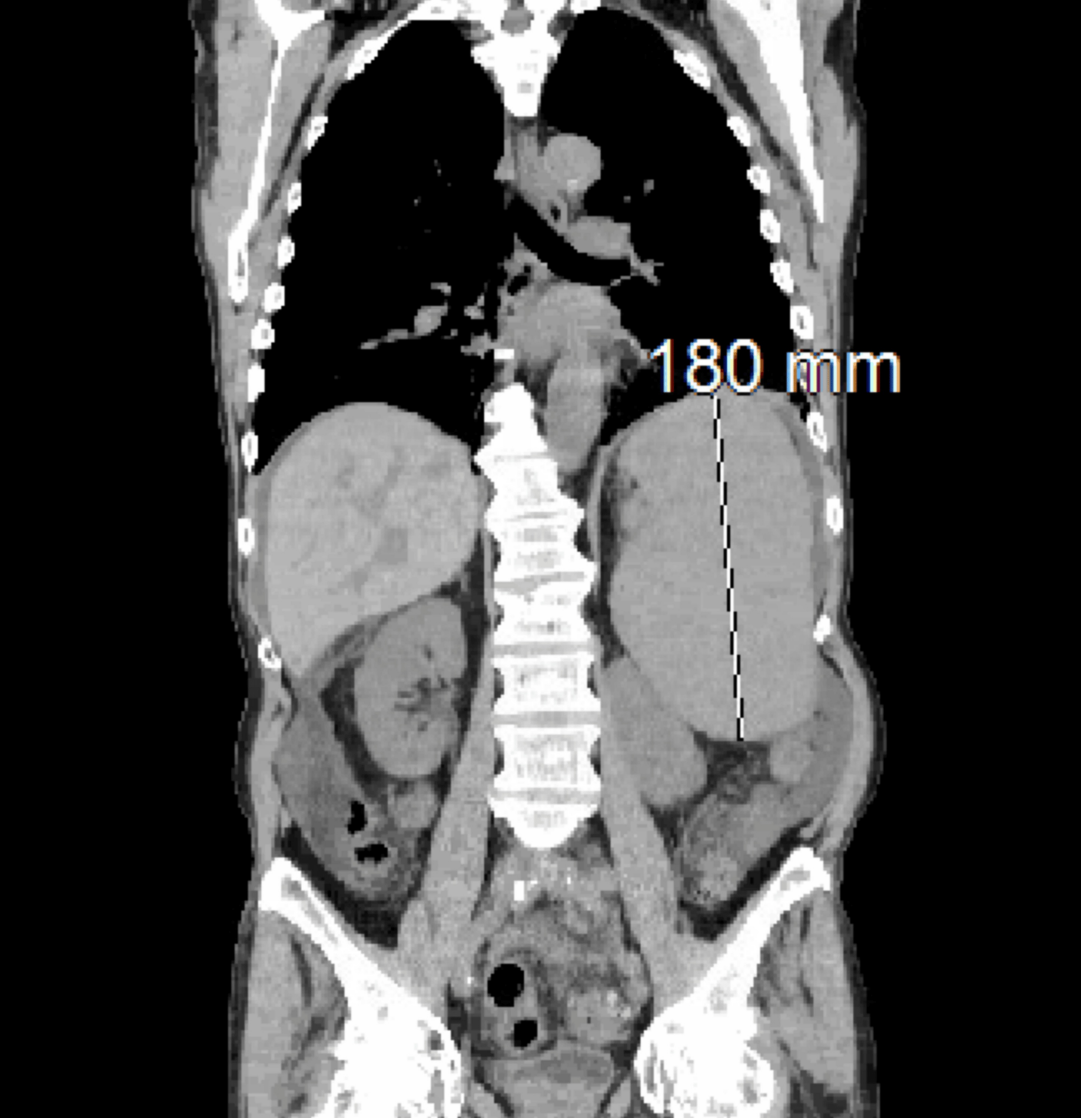
Abdominal CT, June 12, 2014. The abdominal CT reveals splenomegaly with a craniocaudal length of 18 cm, supporting a diagnosis of splenic marginal zone lymphoma.

Abdominal CT revealed an enlarged spleen of length 18 cm (Figure 1), compared to a normal length of 6 to 13 cm [11]. Flow cytometry demonstrated the presence of 16% monoclonal B cells expressing lambda light chains. The monoclonal B cells expressed cluster of differentiation (CD)5 and were negative for CD10 and CD23. An additional fluorescence in situ hybridization (FISH) panel identified deletion of 13q14 and trisomy 12. The patient was diagnosed with splenic MZL and was started on chemotherapy with rituximab and prednisone.

The patient demonstrated a good response to the chemotherapy, showing signs of remission. However, the rituximab was stopped in early 2015 due to complaints of diplopia. Although the diplopia is likely unrelated to rituximab, this symptom was not clearly explained despite a follow-up neurological examination and brain MRI.

In 2019, the patient's blood work showed a creatinine of 1.5 mg/dL, and he was diagnosed with stage 3 chronic kidney disease. Another year later, the creatinine continued to be elevated. However, during these times, the patient was generally feeling better.

**Figure 2:**
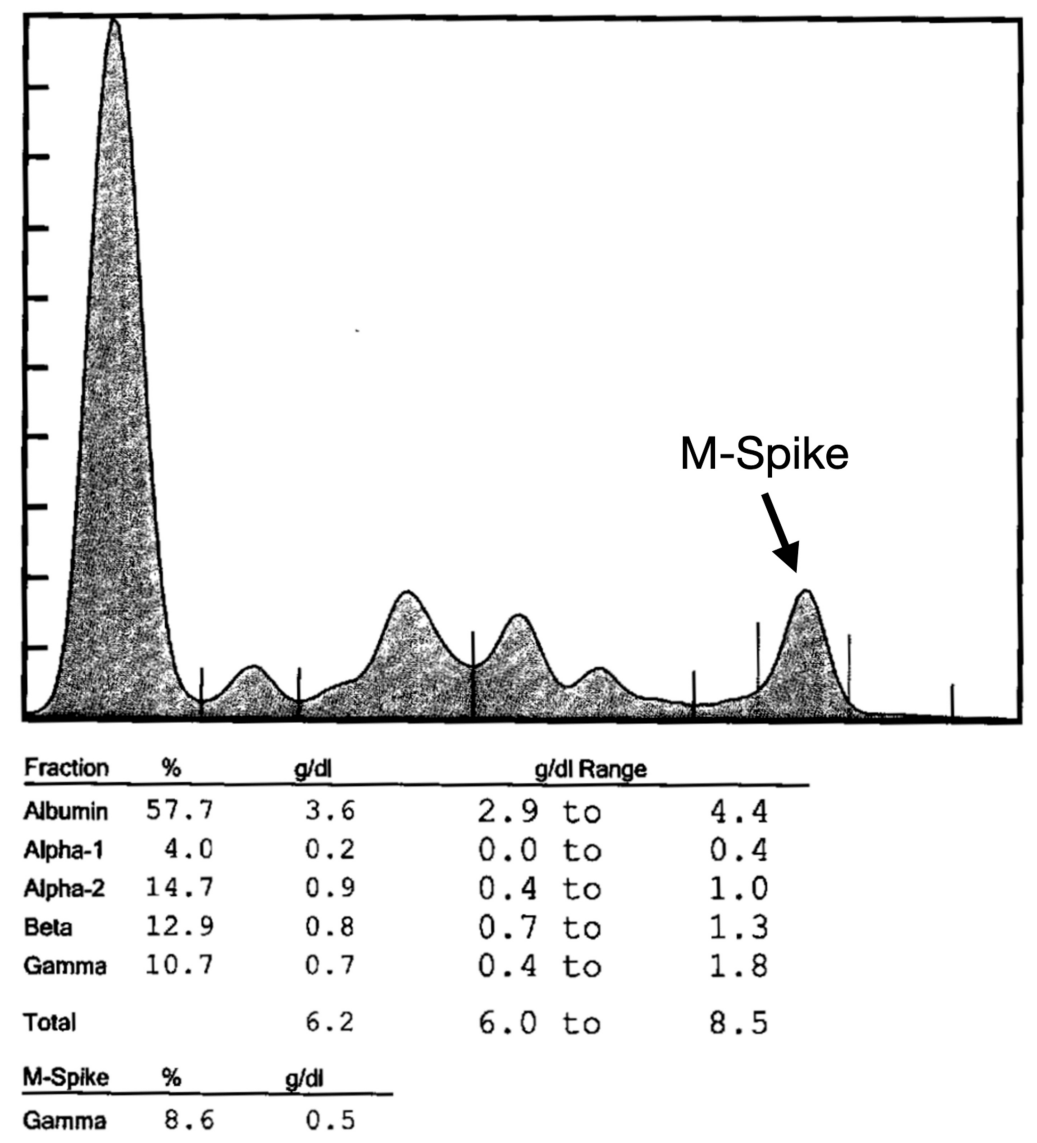
Serum protein electrophoresis (SPEP), February 5, 2021. The SPEP demonstrates an abnormal M-spike finding of 0.5 g/dL, which is normally absent.

In the next year, in 2021, a serum protein electrophoresis was performed, which revealed an M-spike of 0.5 g/dL (Figure 2). Immunofixation electrophoresis was additionally performed, which revealed increased free lambda light chain levels of 465.4 mg/L (normal range 5.7-26.3 mg/L), normal range free kappa light chain levels of 12.5 mg/L (normal range 3.3-19.4 mg/L), and a decreased kappa/lambda ratio of 0.03 (normal range 0.26-1.65). Furthermore, creatinine continued to progress to 1.6 mg/dL. These lab results raised suspicion for multiple myeloma, and a bone marrow biopsy was collected at a different hospital. However, the bone marrow biopsy returned negative results for multiple myeloma. By the end of the year, free lambda light chain levels increased to 954.4 mg/L with a kappa/lambda ratio of 0.01, and creatinine continued to increase to 1.8 mg/dL.

By December 2022, the patient has been feeling lethargic with decreased appetite and weight loss. The patient's creatinine had increased to 5.87 mg/dL, and the free lambda light chain had increased to 1241.3 mg/L. At this point, nephrology was consulted for the management of acute kidney injury and was started on fluids. A renal ultrasound was obtained, which showed bilateral renal parenchymal disease. In January 2023, a renal biopsy was collected and analyzed, which revealed lambda light chain casts, lambda light chain deposition in glomerular capillaries, tubular atrophy, and interstitial fibrosis. These findings resulted in a diagnosis of lambda light chain cast nephropathy.

To address the increased free light chain, which is suspected to be caused by the MZL, the patient was placed on a four-week course of rituximab. However, upon completion of the regimen, the free lambda light chain was only mildly decreased at 1,139.3 mg/L, and the creatinine continued to rise to 6.7 mg/dL. The patient was subsequently switched to zanubrutinib. However, a few months later, the patient's condition deteriorated, and the care plan was transitioned to exploring comfort measures. The patient later expired.

## Discussion

We present a case of lambda light chain cast nephropathy caused by a splenic MZL. The present case is highly unusual, given that the vast majority of cases of light chain cast nephropathy are caused by multiple myeloma.

MZL is an indolent B cell non-Hodgkin lymphoma that develops in the marginal zones of lymphoid tissues, with subtypes including mucosa-associated lymphoid tissue (MALT) lymphomas, splenic MZL, and nodal MZL [12]. MZL has been reported to cause light chain cast nephropathy in only a handful of cases in prior literature. One case is described by Lee et al. (2024) [13], who describe a case of kappa light chain cast nephropathy concurrent with splenic MZL and influenza A infection. The patient reported, in this case, was successfully discharged after hemodialysis and a chemotherapy regime, including bendamustine and rituximab. The more favorable outcome in the case reported by Lee et al. (2024) [13] compared to our case may be partially attributed to the younger age (47 years old) of the patient. A multicenter retrospective study conducted by Martins et al. (2024) [2] identified two patients with extranodal MZL out of 23 patients who developed non-multiple myeloma light chain cast nephropathy.

Several prior works suggest that the presence of the CD5 antigen in MZLs may carry associations with the development of light chain cast nephropathy, as seen in this case. Samarasinghe et al. (2019) [14] report a case of a patient with CD5+ splenic MZL with accelerated chronic kidney disease and IgG kappa light chain restriction. Ferry et al. (1996) [15] report three cases of CD5+ splenic MZL, which all show signs of bone marrow involvement as well as immunoglobulin light chain restriction, including one patient with IgM lambda light chain restriction. Other works have suggested that the presence of CD5 may be a sign of aggressiveness of splenic MZL [16].

Although monoclonal gammopathy is known to be associated with non-Hodgkin lymphomas in general, it appears to be rare in the setting of MZLs [17]. A study by Yamauchi et al. (2019) [18] has found that MALT lymphomas associated with monoclonal gammopathy were more likely to have an aggressive course.

## Conclusions

We have described a rare case of lambda light chain cast nephropathy associated with splenic MZL. This case underscores the importance of considering light chain cast nephropathy in the differential diagnosis of lymphoma patients presenting with renal dysfunction. Key diagnostic evaluations include serum protein electrophoresis, immunofixation electrophoresis, renal ultrasound, and renal biopsy. Treatment should prioritize addressing the underlying lymphoma, such as through chemotherapy, alongside targeted management of renal complications. Further research is warranted to better define prognostic factors predicting the development of paraproteinemia and light chain cast nephropathy in lymphoma patients.
